# Diagnostic Molecular Mycobacteriology in Regions With Low Tuberculosis Endemicity: Combining Real-time PCR Assays for Detection of Multiple Mycobacterial Pathogens With Line Probe Assays for Identification of Resistance Mutations

**DOI:** 10.1016/j.ebiom.2016.06.016

**Published:** 2016-06-14

**Authors:** Vanessa Deggim-Messmer, Guido V. Bloemberg, Claudia Ritter, Antje Voit, Rico Hömke, Peter M. Keller, Erik C. Böttger

**Affiliations:** a Institut für Medizinische Mikrobiologie, Universität Zürich, Gloriastrasse 30/32, 8006 Zürich, Switzerland; b Nationales Zentrum für Mykobakterien, Gloriastrasse 30/32, 8006 Zürich, Switzerland

**Keywords:** ATS, American Thoracic Society, CP, crossing point), CSF, cerebrospinal fluid, DOTS, directly observed treatment, short-course, DST, drug susceptibility testing, EMB, ethambutol, IDSA, Infectious Diseases Society of America, INH, isoniazid, LPA, line probe assay, MDR, multidrug resistant, MTB, *Mycobacterium tuberculosis* complex, NAT, nucleic acid testing, NPV, negative predictive value, NTM, nontuberculous mycobacteria, PCR, polymerase chain reaction, PPV, positive predictive value, PZA, pyrazinamide, QDST, quantitative drug susceptibility testing, RIF, rifampicin, SDS, sodium dodecyl sulfate, TB, tuberculosis, WHO, World Health Organization, XDR, extremely drug resistant, Real-time PCR, Line probe assay, *Mycobacterium tuberculosis*, Nontuberculous mycobacteria, Drug susceptibility

## Abstract

Molecular assays have not yet been able to replace time-consuming culture-based methods in clinical mycobacteriology.

Using 6875 clinical samples and a study period of 35 months we evaluated the use of PCR-based assays to establish a diagnostic workflow with a fast time-to-result of 1–2 days, for 1. detection of *Mycobacterium tuberculosis* complex (MTB), 2. detection and identification of nontuberculous mycobacteria (NTM), and 3. identification of drug susceptible MTB.

MTB molecular-based detection and culture gave concordant results for 97.7% of the specimens. NTM PCR-based detection and culture gave concordant results for 97.0% of the specimens. Defining specimens on the basis of combined laboratory data as true positives or negatives with discrepant results resolved by clinical chart reviews, we calculated sensitivity, specificity, PPV and NPV for PCR-based MTB detection as 84.7%, 100%, 100%, and 98.7%; the corresponding values for culture-based MTB detection were 86.3%, 100%, 100%, and 98.8%. PCR-based detection of NTM had a sensitivity of 84.7% compared to 78.0% of that of culture-based NTM detection. Molecular drug susceptibility testing (DST) by line-probe assay was found to predict phenotypic DST results in MTB with excellent accuracy.

Our findings suggest a diagnostic algorithm to largely replace lengthy culture-based techniques by rapid molecular-based methods.

## Highlights

• Molecular assays have not yet been able to replace time-consuming culture-based methods in the mycobacteriology laboratory.
• We have evaluated genetic tests for: i) detection of MTB, ii) detection of NTM, and iii) identification of susceptible MTB.
• Our findings suggest a diagnostic algorithm to replace lengthy culture-based techniques by rapid molecular-based methods.

There are > 700 reports on molecular detection of tuberculosis in respiratory and non-respiratory specimens. Limited published data exist on molecular tests for detection of nontuberculous mycobacteria (NTM) and tuberculosis drug susceptibility in clinical samples. We demonstrate an excellent accuracy of molecular-based detection of tuberculosis and NTM in conjunction with molecular-based rapid recognition of drug-susceptible and drug-resistant tuberculosis. The diagnostic algorithm developed in this work allows the rapid recognition of clinically relevant mycobacterial infections and tuberculosis drug resistance.

## 1 Introduction

The genus *Mycobacterium* is separated into the pathogenic species *Mycobacterium tuberculosis* complex, *Mycobacterium leprae*, and *Mycobacterium ulcerans*, and various nontuberculous mycobacteria (NTM) (Manual of Clinical Microbiology, 2015).

*Mycobacterium tuberculosis* (MTB) is the causative agent of tuberculosis. Global priorities for tuberculosis (TB) care and control are to improve case-detection and to enhance rapid diagnosis of tuberculosis disease (WHO, 2014). Directly observed standardized short-course chemotherapy (DOTS) is an effective treatment for drug-susceptible tuberculosis disease, with cure rates > 95.0% (Hopewell et al., 2006). However, the current situation is characterized by increasing numbers of drug-resistant tuberculosis disease. The treatment of multidrug-resistant (MDR) or extensively drug-resistant (XDR) TB requires the use of second-line drugs, which are less effective, more expensive, and more toxic than first-line drugs (WHO, 2014, Hopewell et al., 2006).

NTM are ubiquitously present in the environment. Their pathogenic potential is variable and infections predominantly occur in patients with underlying disorders or immunocompromising conditions, although individuals without predisposing risk factors can also be affected (Griffith et al., 2007, Aksamit et al., 2014). Diseases caused by NTM include lung disease (e.g. *Mycobacterium abscessus*, *Mycobacterium kansasii*), cutaneous ulcers (e.g. *Mycobacterium marinum*, *M. ulcerans*), disseminated infections (e.g. *Mycobacterium genavense*), lymphadenitis (e.g. *Mycobacterium avium*), and joint infections (e.g. *Mycobacterium haemophilum*) (Tortoli, 2009, Tortoli, 2014). The variety of diseases caused by NTM, the association of NTM infections with underlying disorders such as bronchiectasis and severe immunosuppressive conditions, and the difficulty to distinguish NTM pulmonary infections from MTB pulmonary infections on the basis of clinical symptoms and imaging alone, all of these underline the need for detection and proper identification of NTM in addition to that of MTB in clinical specimens (Griffith et al., 2007).

Laboratory diagnosis of mycobacterial infections is done in designated and specialized laboratories not the least due to the need for biosafety conditions, and the same isolation procedures and cultural techniques are used for recovery of *M. tuberculosis* and all of the NTM pathogens. Traditionally, detection of mycobacteria and drug susceptibility testing (DST) is based on culture, which is a time consuming, complicated, resource costly and lengthy process. Recovery of mycobacteria by culture and identification takes about 2–4 weeks, followed by an additional 1–2 weeks for drug susceptibility testing (Manual of Clinical Microbiology, 2015).

PCR-based diagnostic procedures for direct detection of MTB in clinical specimens have been introduced > 20 years ago (Boddinghaus et al., 1990, Thierry et al., 1990). A number of genetic assays for detection of MTB DNA in clinical specimens are commercially available, e.g. AMTD Amplified Mycobacterium Tuberculosis Direct Test (Gen-Probe Inc., San Diego, USA), COBAS™ TaqMan™ MTB test (Roche, Basel, Switzerland), BD ProbeTec ET system (Becton Dickinson, Baltimore, USA), Abbott LCx *M. tuberculosis* assay (Abbott Laboratories, Chicago, USA), and Fluorotype MTB (Hain Lifescience GmbH, Nehren, Germany), as are numerous studies comparing molecular and culture-based methods for detection of MTB (Reischl et al., 1998, Goessens et al., 2005, Kim et al., 2011, Tortoli et al., 2012, Bloemberg et al., 2013, Hofmann-Thiel and Hoffmann, 2014). In contrast to MTB, molecular genetic assays for detection of NTM are only infrequently implemented in routine diagnostics, although various in-house assays have been described (Kirschner et al., 1996, Del Portillo et al., 1996, Stauffer et al., 1998, Shrestha et al., 2003, Peter-Getzlaff et al., 2010).

Different formats have been developed for detection of resistance conferring mutations in MTB. Compared to the GeneXpert MTB/RIF assay (Boehme et al., 2010) which is limited to the detection of rifampicin resistance, line probe assays are particularly attractive due to their unmatched versatility, i.e., their ability to detect multiple mutations in different chromosomal targets, and their low cost (Hillemann et al., 2005, Hillemann et al., 2009, Barnard et al., 2012, Jacobson et al., 2013, Ritter et al., 2014, Domínguez et al., 2016). Several line probe assays are commercially available, e.g. GenoType MTBDRsl (Hain Lifescience GmbH, Nehren, Germany), AID TB Resistance (AID Diagnostika GmbH, Strassberg, Germany), and INNO-LiPA Rif TB (Fujirebio Europe N.V., Gent, Belgium).

More recently, WHO recommended the implementation of molecular-based tests for diagnosis of infections with MTB and to rapidly detect drug resistant tuberculosis (WHO, 2008, WHO, 2011, WHO, 2013). In regions with low *M. tuberculosis* endemicity the nearly exclusive focus of commercially available assays on MTB with largely neglecting NTM has slowed down the implementation of molecular-based diagnostics for replacement of culture in diagnostic mycobacteriology. Any strategy to replace culture by molecular detection assays in countries with low *Mycobacterium tuberculosis* endemicity has to address both MTB and NTM and to include screening for susceptibility to first-line TB drugs. Switzerland, the country where this study was conducted, is a low prevalence region, with TB case rates of 6.6/100.000 in 2012 and < 2% MDR/XDR (Somoskövi et al., 2014). The purpose of this study was to compare the performance of a combination of molecular assays for detection of MTB, NTM and mutations associated with drug resistance in MTB with that of culture-based methods.

## 2 Material and Methods

### 2.1 Clinical Samples

**Fig. 1:**
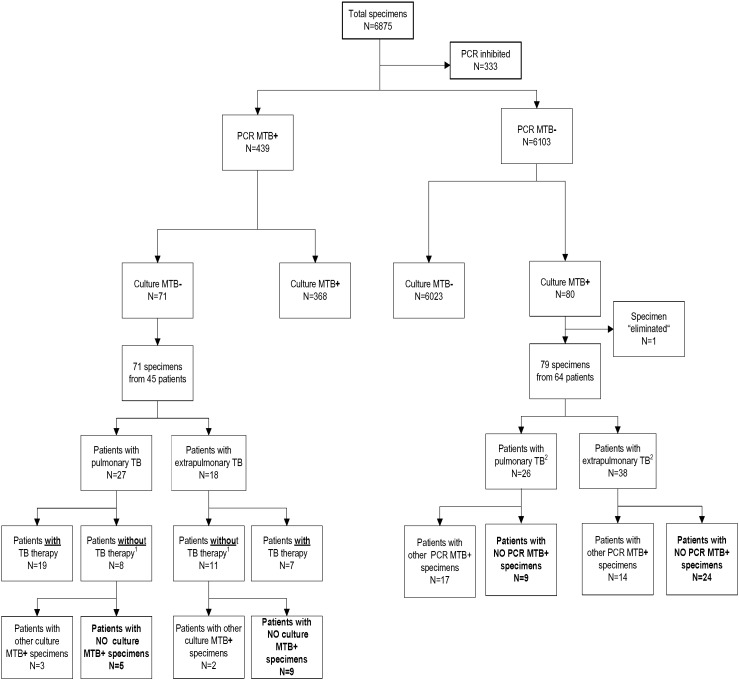
Workflow of clinical specimens studied using the COBAS™ TaqMan™ MTB assay. Abbreviations: MTB: *Mycobacterium tuberculosis*; TB: tuberculosis. ^1^For details: see Table S4 in the Supplemental materials. ^2^For details: see Table S5 in the Supplemental materials.

All samples submitted to the Institute of Medical Microbiology, University of Zurich, for molecular detection of MTB or NTM were included in this study. The study went over a period of 35 months and consisted of 6875 clinical specimens (4928 respiratory and 1947 non-respiratory specimens) originating from 3384 patients (Fig. 1). The 4928 respiratory specimens consisted of sputum, n = 2917; tracheal bronchial aspirate, n = 1145; bronchial alveolar lavage fluid, n = 724; bronchial aspirate, n = 110; and others, n = 32. The 1947 non-respiratory specimens consisted of aspirate, n = 663; tissue, n = 420; urine, n = 212; cerebrospinal fluid (CSF), n = 192; biopsy n = 82; wound swab, n = 75; lymph node, n = 83; gastric fluid, n = 34; bone marrow, n = 43; ascites, n = 36; abscess, n = 20; stool n = 20; blood n = 29; non-specified, n = 10; bone, n = 9; ejaculate, n = 2; and others, n = 17. Consistently, all samples were analyzed in parallel using culture-based procedures and a modified COBAS™ TaqMan™ MTB test (Roche, Basel, Switzerland) for direct detection of MTB and NTM. MTB PCR positive specimens were screened for mutations associated with resistance to isoniazid (INH) and rifampicin (RIF) using the AID TB resistance line probe assay module 1 (AID Diagnostika GmbH, Strassberg, Germany). If resistance mutations were detected by LPA module 1, LPA modules 2 (streptomycin, amikacin, capreomycin) and 3 (fluoroquinolones, ethambutol) were used for screening for mutations associated with resistance to second-line drugs. At the time, this study was conducted, no ethical approval was required to conduct a non-interventional laboratory-based study.

### 2.2 Decontamination of Specimens, Microscopy, Culture, and Phenotypic Drug Susceptibility Testing

Clinical specimens, with the exception of cerebrospinal fluid (CSF) and bone marrow were decontaminated using the *N*-acetyl-l-cysteine-sodium hydroxide method. CSF and bone marrow were decontaminated using the SDS-NaOH method (Manual of Clinical Microbiology, 2015). The resuspended sediment was used to prepare auramine-rhodamine stained smears and to inoculate MGIT 960 liquid broth medium (Becton Dickinson, Towson, MD) and Middlebrook 7H11 plate media for growth detection by incubation at 37 °C for a maximum of 7 weeks. Positive auramine-rhodamine microscopy results were confirmed by Ziehl-Neelsen staining. Positive cultures were identified to species level by 16S rRNA gene sequence analysis as described previously (see below). Phenotypic drug susceptibility testing to first-line TB agents (isoniazid, rifampicin, ethambutol, pyrazinamide) was done for all MTB isolates by using the MGIT 960 system as recommended by the manufacturer (Becton Dickinson, East Rutherford, NJ, USA). Drug resistant MTB isolates were subsequently analyzed in detail by quantitative drug susceptibility testing (QDST) using the MGIT 960 system and the EpiCenter™ software equipped with the TBeXiST™ module as described previously (Springer et al., 2009, Cambau et al., 2015).

### 2.3 Detection of Mycobacterial DNA

DNA was extracted from decontaminated samples using the respiratory specimen kit (Roche Diagnostics). The COBAS™ TaqMan™ MTB test was performed according to the manufacturer's instructions. A sample was categorized as positive when a PCR crossing point (CP) < 45 was registered (Diagnostics, 2010). When no other MTB positive sample was recorded of a patient, a second DNA extract of the original specimen was tested for confirmation. In case the second extract was tested negative, culture was negative, smear was negative and no further clinical information of the patient was available, the specimen was reclassified as PCR negative. In our study only one such PCR positive specimen (a sputum) was reclassified as negative.

Based on previous studies we developed a *Mycobacterium* genus probe for detection of NTM DNA in clinical specimens using the COBAS™ TaqMan™ platform (Peter-Getzlaff et al., 2010). A *Mycobacterium* genus TaqMan™ probe was designed (5′-Cy5-CGACAAACCACCTACGAGCTCTTTACGC-BBQ-3′) and added (1 μl of a 10 μM stock solution) to the reaction mixture. The genus probe was designed from a multiple sequence alignment of all known mycobacterial 16S rRNA gene sequences deposited with NCBI Genbank using Lasergene DNAstar MegAlign software version 7. For the detection of genus probe hybridization we used the open channel mode of the COBAS™ TaqMan™ MTB assay. For samples positive in the *Mycobacterium* genus assay (CP < 45) the PCR amplicon was sequenced for species identification. The *Mycobacterium* genus probe did not affect the sensitivity of the MTB probe and was able to detect a wide range of fast- and slow-growing mycobacteria (Tables S1, S2 and S3 in Supplemental Materials).

### 2.4 Amplification, DNA Purification, and DNA Sequencing of Positive Samples and Cultures

Amplicons with a CP ≥ 45 or a maximum curve value < 0.5 in the COBAS™ TaqMan™ MTB assay and samples that scored positive in the *Mycobacterium* genus assay (CP < 45) were purified using the QIAquick PCR purification Kit (Qiagen, Hombrechtikon, Switzerland) and subjected to 16S rRNA gene sequencing using primer Mbakt-14 (5′-GRG RTA CTCGAG TGG CGA AC-3′) (Bosshard et al., 2006). SmartGene IDNS software and databases (SmartGene GmbH, Zug, Switzerland) were used for sequence analysis. Homology analysis and species identification were carried out as described previously (Springer et al., 1996).

Sequencing of the 16S rRNA gene from cultured isolates was done using primers 283 and 264 for PCR amplification and primer Mbakt-14 for sequencing. For cultured isolates, further sequence analysis of the *rpoB* gene was used to distinguish between closely related species within the *Mycobacterium chelonae*/*abscessus* complex or within the *Mycobacterium fortuitum* complex (Adékambi et al., 2003); sequence analysis of *hsp65* was used for *M. kansasii*/*gastri* differentiation, and sequence analysis of the 3′ end of the 16S rRNA gene was used to differentiate *M. marinum* from *M. ulcerans* (Hofer et al., 1993).

### 2.5 Molecular Detection of MTB Resistance Mutations

DNA extracts of samples positive in the COBAS™ TaqMan™ MTB assay were subjected to further analyses to identify mutations in the *rpoB*, *inhA* and *katG* genes conferring resistance to isoniazid and rifampicin, respectively. The AID TB reverse hybridization line probe assay (AID Diagnostika GmbH, Strassberg, Germany) was performed according to the manufacturer's instructions (Ritter et al., 2014). This LPA assay detects specific mutations in *inhA* promoter (positions − 16, − 15, − 8), in *katG* (codon 315) and in *rpoB* (codons 516, 526, 531). The absence of hybridization of a wild-type probe and/or the presence of hybridization of a mutated gene probe suggests the presence of a genotypic resistance mutation. When any mutation in *rpoB*, *inhA*, and/or *katG* was detected, the specimen was subsequently tested with AID TB resistance LPAs module 2 (streptomycin, amikacin, capreomycin) and module 3 (fluoroquinolones, ethambutol) (Ritter et al., 2014).

LPA module 2 contains probes for detection of streptomycin resistance (*rpsL* mutations K43R, K88R, K88Q) and *rrs* mutations C513T, A514C, G515C, and C517T (corresponding to *Escherichia* *coli* *rrs* positions 523, 524, 525, and 527, respectively), and for detection of amikacin or capreomycin resistance (*rrs* mutations A1401G, C1402T, and G1484C/T, corresponding to *E. coli rrs* positions 1408, 1409, and 1491, respectively). LPA module 3 contains probes for detection of fluoroquinolone resistance (*gyrA* A90V, S91P, D94A, D94N, D94Y, and D94G) and for detection of ethambutol resistance (*embB* M306V and M306I). Each line probe module contained a *M. tuberculosis* probe as positive control and a human GAPDH probe as amplification control. Results of the line probe assay were scored as uninterpretable when the positive MTB control did not show up or when high background signals were observed.

### 2.6 Analysis of Molecular Results

The results of the genetic assays were compared to culture and phenotypic DST. In case of discrepancy, clinical data as well as laboratory data from additional patient specimens were taken into consideration. For discrepant results in module 1 versus phenotypic DST (isoniazid and rifampicin) the target genes *inhA* promoter, *inhA* structural gene, *katG* and *rpoB* were sequenced.

### 2.7 Case Definitions

For the purpose of the study the definitions are as follows:

1. Patients were defined as MTB positive when MTB culture and MTB PCR were positive. Patients were scored MTB negative when all specimens were negative by culture and PCR. In case of discrepant culture/PCR results patients' clinical data were reviewed to reach a firm diagnosis. On basis of this definition sensitivity, specificity, positive predictive value and negative predictive value were calculated.
2. NTM infections were identified by a positive NTM culture and/or *Mycobacterium* genus positive/MTB negative PCR, if DNA sequence analysis revealed a pathogenic NTM. Clinical data were reviewed and together with laboratory results from additional specimens were used to resolve discrepant culture/PCR results and to establish the diagnosis of NTM infection supported by criteria as defined by the ATS guidelines (Griffith et al., 2007). As inherent to a genus probe, this probe also detects non-pathogenic NTM and species of closely related genera, such as *Corynebacterium* (Peter-Getzlaff et al., 2010). Patients were scored NTM negative when culture and the *Mycobacterium* genus PCR were negative or sequence analysis failed to identify a pathogenic NTM. We used broadly cited literature to classify NTM as pathogenic and non-pathogenic (Table 1) (Manual of Clinical Microbiology, 2015).
3. DNA extracts of MTB PCR positive specimens were tested for the presence of mutations associated with resistance to isoniazid and rifampicin using the AID TB resistance LPA module 1. When any mutation was detected, the specimen was categorized as drug resistant. When only wild-type probes were detected the isolate was categorized as drug susceptible. For phenotypic drug susceptibility testing, susceptibility was defined by the critical concentration (Springer et al., 2009).

### 2.8 Statistics

The 2 × 2 contingency table was used to calculate sensitivity, specificity, positive and negative predictive value. Cohen's kappa and chi-squared tests were applied for calculating agreement and significance in differences between results of different methods (Pagano and Gavreau, 2000). The required study sample size to reach a confidence level of 95% and a confidence interval of 0.5 was calculated to be 5921 samples using Survey Software (Creative Research Systems, Sebastopol, CA, USA).

### 2.9 Role of the Funding Source

The funders of the study had no role in study design, data collection, data analysis, data interpretation, or writing of the report. The corresponding author had full access to all data in the study and had final responsibility for the decision to submit for publication.

## 3 Results

During the 35-months period a total of 6875 respiratory and non-respiratory specimens from 3384 patients were submitted to the mycobacteriology laboratory for mycobacterial culture and PCR (Fig. S1 in Supplemental materials provides a flow diagram on processing of the clinical specimens).

### 3.1 Detection of MTB by the COBAS™ TaqMan™ MTB Assay and Comparison to Culture

The COBAS™ TaqMan™ MTB PCR was inhibited in 333/6875 (4.8%) specimens, negative in 6103/6875 (88.8%) specimens, and positive in 439/6875 (6.4%) specimens. Overall, MTB PCR and culture showed 97.7% concordant results (Fig. 1).

For the purpose of overall analysis we defined a patient as MTB positive when MTB culture and MTB PCR were positive. Patients were scored MTB negative when all specimens were negative by PCR and culture. In the case of discrepant culture/PCR results, patients' clinical data were reviewed and test results from additional samples considered to reach a firm diagnosis. Discrepant test results were observed for 150 samples (71 PCR MTB pos., culture MTB neg., 25/71 specimens originated from patients without prior or ongoing TB therapy; 79 PCR MTB neg., culture MTB pos.) corresponding to a total of 109 patients (Fig. 1). On the basis of the results of parallel samples and clinical chart reviews (Tables S4 and S5 in Supplemental Materials) none of the positive PCR or positive culture results was judged as false positive. Overall sensitivity, specificity, PPV and NPV calculated for culture-based MTB detection were 86.3%, 100%, 100%, 98.8%, and for PCR-based MTB detection 84.7%, 100%, 100%, and 98.7%, respectively. Comparison of culture and PCR results showed that for pulmonary TB, 5 patients would have been missed by culture and 9 patients would have been missed by PCR. For extrapulmonary TB, 9 patients would have been missed by culture and 24 by PCR. Statistical analysis using the chi-square test showed that these differences were not significant [for pulmonary TB, χ^2^ (1, N = 34) = 1.964, *p* = *0*.*161*, for extra pulmonary TB, χ^2^ (1, N = 49) = 0.1351, *p* = *0*.*245*].

### 3.2 Detection of NTM DNA by the Modified COBAS™ TaqMan™ MTB Assay and Comparison to Culture

**Fig. 2:**
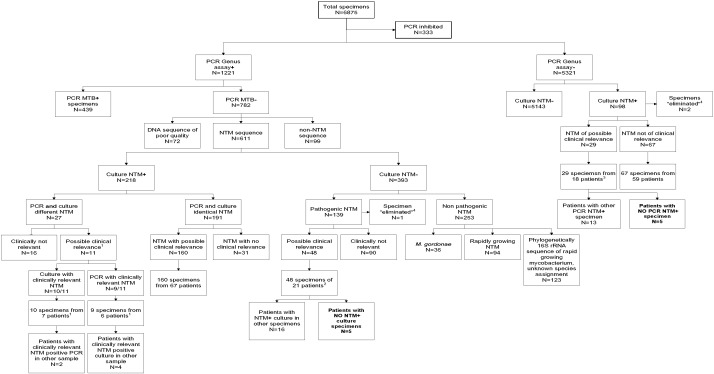
Workflow of clinical specimens studied using the COBAS™ TaqMan™ *Mycobacterium* genus assay. Abbreviations: MTB: *Mycobacterium tuberculosis*; NTM: nontuberculous mycobacteria. ^1^For details: see Table S6 in the Supplemental materials; in total 8 patients, 7/8 patients had a culture with a clinically relevant NTM and 6/8 patients had a PCR with a clinically relevant NTM. ^2^For details: see Table S7 in the Supplemental materials. ^3^For details: see Table S8 in the Supplemental materials. ^4^Specimen eliminated due to lacking clinical information.

Using the modified COBAS™ TaqMan™ MTB assay, 1221/6875 (17.8%) clinical specimens tested positive with the *Mycobacterium* genus probe (Fig. 2). A total of 782/1221 (64.0%) specimens tested positive with the *Mycobacterium* genus probe but were negative with the MTB probe, pointing to the presence of NTM DNA. 16S rRNA gene amplicon sequence analysis was performed on all 782 *Mycobacterium* genus positive/MTB negative PCRs. For 99/782 (12.7%) samples, DNA sequencing revealed species of closely related genera, such as *Corynebacterium*, for 72/782 (9.2%) samples no readable sequence was obtained. All of these 171 samples were negative for NTM by culture.

In 611/782 (78.1%) specimens NTM was identified to species level. For 218/611 (35.7%) specimens at least one NTM species was also recovered by culture. In 191/218 (87.6%) specimens (107 smear positive) the 16S rRNA gene sequence of the NTM recovered by culture was identical to the DNA sequence of the PCR amplicon obtained from the clinical specimens (Table 1). 160 of the 191 NTM specimens (corresponding to 67 patients) were considered to be clinically significant. In 27/218 NTM culture positive specimens, the NTM recovered by culture was different from the NTM identified by PCR (Fig. 2). Evaluation of the patients' clinical history showed that for 11/27 specimens (8 patients) a clinical relevant NTM was identified by PCR and/or culture (Table S6 in Supplemental materials).

For 393/611 *Mycobacterium* genus PCR positive specimens with proper NTM sequence identification, culture was negative for NTM (Fig. 2). For 253/393 (64.6%) specimens the NTM sequence identified corresponded either to a fast growing NTM considered an environmental contamination or to a species with no clinical relevance (*Mycobacterium gordonae*). 130/253 16S rRNA sequences were assigned to a defined NTM species (Table 1), 123/253 sequences could only be assigned to the phylogenetic branch of rapidly growing NTMs with no further species identification possible as a corresponding sequence was not found in the database (Springer et al., 1996). Presumably, these sequences represent environmental contaminants reflecting unknown rapid growing mycobacteria. In 139/393 (35.4%) PCR NTM positive and culture negative specimens, the identified NTM sequence corresponded to what was considered a pathogenic NTM (Table 1). For 5/139 specimens, analysis of the 16S rRNA sequence allowed an assignment to the branch of slow growing mycobacterial species, but could not be further differentiated and presumably correspond to potentially novel species (Tortoli, 2014). After review of the patients' clinical charts, we conclude that 48/139 samples, obtained from 21 patients (19 respiratory diseases, 2 nonrespiratory disease) with a pathogenic NTM species identified by NTM PCR but a negative culture, were suggestive of a mycobacterial infection and clinically significant (Table S7 in Supplemental materials). In 5/22 patients no other specimen from the same patient recovered the respective pathogen by culture. For 4/5 patients with negative cultures, 3 or more specimens had been submitted for culture (Table S7 in Supplemental materials).

A total of 5321 specimens tested negative in the COBAS™ TaqMan™ *Mycobacterium* genus assay. 98/5321 (1.8%) specimens obtained from 78 patients were NTM culture positive with one or two NTMs (Table 1), no species assignment could be made for one NTM isolate on basis of the 16S rRNA gene sequence. Analysis of the patients' clinical history indicated that 29/98 NTM isolates recovered were of clinical significance; the 29 samples correspond to 18 patients (14 respiratory disease, 4 non respiratory disease), 2 of which were smear microscopy positive (Table S8 in Supplemental Materials). For 5/18 patients the cultured NTM was not detected by PCR in any equivalent specimen analyzed.

Overall, NTM PCR and culture showed 97.0% concordant results for (potential) clinically relevant NTM (Fig. 2). Statistical analysis using the chi-square test showed that the number of missed NTM patients by culture (n = 5) or PCR (n = 5) were not significantly different [χ^2^ (1, N = 40) = 0.135, *p* = *0*.*714*].

### 3.3 Direct Detection of Mutations Associated With Drug Resistance Using the AID TB Resistance Line Probe Assay and Comparison to Phenotypic DST

In total, 438/439 PCR MTB positive specimens were analyzed by AID TB resistance LPA (Table 2). The specimens consisted of 324 (73.9%) respiratory and 114 (26.0%) non-respiratory specimens. 311/438 specimens (71.0%) showed an interpretable result. Overall, 78.7% of the respiratory and 49.1% of the non-respiratory specimens showed an interpretable result. Of the 127 specimens with an uninterpretable result, 104 (81.9%) were smear-negative (58 respiratory specimen, 46 non-respiratory specimens) and 23 (18.1%) were smear-positive (11 respiratory specimens, 12 non-respiratory specimens). 91.7% of the smear positive samples gave an interpretable line probe result, as compared to only 35.0% for smear negative samples.

The 438 specimens analyzed by the AID TB resistance LPA assay were obtained from 253 patients (Table 3). For 195/253 (77.1%) patients at least one specimen showed an interpretable result. For 58/253 (22.9%) patients only uninterpretable LPA results were obtained (Table 3). Calculating the percentage of uninterpretable results at patient level revealed this to be clearly dependent on the number of samples analyzed. 39/58 (67.2%) patients with an uninterpretable LPA corresponded to patients for which only one specimen was tested, 14/58 (24.1%) patients with an uninterpretable LPA originated from patients for which a total of 2 specimens were analyzed and only for 5/58 (8.6%) patients with an uninterpretable LPA ≥ 3 clinical specimens were tested.

For 239/253 (94.5%) patients at least one culture grew MTB allowing phenotypic DST to be done. For the 58/253 patients with no interpretable LPA result, cultured DST was available for 56/58 patients – 54 patients had fully drug susceptible TB, 2 patients had monoresistance to isoniazid (1 low-level INH resistance associated with an *inhA*-15 promoter mutation, 1 high-level resistance associated with a *katG* S315T mutation). Phenotypic DST was available for 183/195 (93.8%) patients with an interpretable LPA result, allowing for a comparison of LPA from clinical specimens and phenotypic DST from culture. 174/183 (95.1%) patients had wild-type test results for *inhA*, *katG* and *rpoB* in the LPA assay indicating fully drug susceptible tuberculosis. This was corroborated by phenotypic DST for 172/174 (98.9%) cultured isolates (Table 4). 2/174 (1.1%) patients with wild-type pattern LPA showed low-level resistance to INH (resistant at 0.1 mg/l, susceptible at 1.0 mg/l; Table S9 in Supplemental Materials). Sequencing of *katG*, *inhA* structural gene and *inhA* promoter from the corresponding isolates did not reveal any mutation. 9/183 (4.9%) patients had a mutation in one or several of the target genes: 5 patients had a mutation in *inhA* (n = 1) or *katG* (n = 4), 4 patients showed mutations in *rpoB* and *katG*, indicating MDR. The phenotypic DST results were in full concordance with the mutational analysis, including one specimen with a mutation at *rpoB* codon 526 as indicated by failing hybridisation of the wild-type probe in the LPA assay. Subsequent sequence analysis of the corresponding cultural isolate revealed a His526Leu alteration (Table S10 in Supplemental Materials). In one case, LPA missed RMP resistance. However, as per the algorithm, this resistant isolate would not have been missed due to the simultaneous presence of a *katG* S315 T mutation. The strain showed a 9 base pair deletion at codons 509–511 by *rpoB* gene sequencing (Table S9 in Supplemental Materials). Overall, LPA and phenotypic DST results for INH and RMP were congruent for 180/183 (98.4%) patients (Table 4). Comparison of RIF and INH molecular screening results with phenotypic DST results shows a high Cohen's kappa of agreement, i.e., κ 0.886 and κ 0.894, respectively.

AID TB resistance LPA modules 2 or 3 were done on all clinical specimens which showed a mutation in LPA module 1 (n = 9). As per the genetic analysis 0/9 specimens was pre-XDR or XDR (*gyrA* probe was wild-type, *rrs* probes 1401, 1402 and 1484 were wild-type). Mutations associated with streptomycin resistance were detected in 5/9 specimens, 3/9 specimens showed mutations associated with ethambutol resistance. One isolate showed a mutation in *embB* (codon M306 V) but was susceptible to ethambutol at the critical concentration of 5 mg/l. Two isolates were resistant to streptomycin at the critical concentration of 1 mg/l, but by LPA had no identifiable mutation in the *rpsL* or *rrs* target gene. For a complete overview of the LPA results and QDST on these isolates see Table S10 in the Supplemental materials.

## 4 Discussion

Conventional methods for detection of mycobacteria include smear microscopy (AFB) and isolation by culture followed by phenotypic DST. However, smear microscopy lacks both sensitivity and specificity, and culture-based detection methods are time-consuming with a turnaround time of 2–4 weeks for initial culture and an additional 1–2 weeks for phenotypic drug susceptibility testing of first-line TB drugs (Manual of Clinical Microbiology, 2015). Nucleic acid-based detection of mycobacteria and identification of resistance mutations by PCR based assays significantly shorten overall time-to-result to 1–2 days. Although molecular based methods are associated with substantial costs per test, these methods may in principle reduce the need for costly high-safety laboratories. In addition, the shortening of time-to-result has major implications in patient management, thereby reducing health care-associated costs.

In the first part of our study we evaluated the performance of PCR based detection of *M. tuberculosis* in patients' specimens in comparison to culture (Fig. 1). Discrepant culture/PCR results were resolved by evaluating clinical data, the patients' clinical history and the availability of additional patient specimens that were analyzed by culture and PCR. The final outcome of these analyses shows that for pulmonary TB disease 5 patients would have been missed by culture alone and 9 patients would have been missed by PCR alone. Statistical analysis showed that this difference is not significant (*p* > 0.05), testifying that PCR and culture have a similar sensitivity in detecting *Mycobacterium tuberculosis* in patients with pulmonary TB. For extrapulmonary TB, 9 patients were missed by culture and 24 patients were missed by PCR. Detection of extrapulmonary TB is often limited by the availability of < 3 specimens per patient. Using the chi-square test the difference between PCR and culture-based detection of NTM was shown not to be significant, indicating a similar sensitivity of PCR and culture for diagnosing extrapulmonary TB. The overall sensitivity, specificity, PPV, and NPV at specimen level (including respiratory and non-respiratory specimens) were calculated for culture-based MTB detection as 86.3%, 100%, 100%, and 98.8%; the corresponding values for PCR-based MTB detection were 84.7%, 100%, 100%, and 98.7%.

The second part of this study evaluated the detection of NTM in clinical specimens by PCR in comparison to culture (Fig. 2). Here we used the Roche COBAS™ MTB assay supplemented with an additional pan *Mycobacterium* genus probe. The *Mycobacterium* genus probe is highly sensitive but compromised by limited specificity requiring subsequent confirmation testing, i.e., sequence analysis of the PCR amplicon. Due to its high sensitivity, the *Mycobacterium* genus probe readily detects contaminating DNA from environmental NTM present in laboratory chemicals and/or non-sterile specimens (especially sputum). The large number of NTM PCR positive specimens which were negative by culture (n = 393, of which n = 139 represent pathogenic NTM) is in part explained by the optimization of established cultural procedures towards recovery of MTB rather than NTM (Manual of Clinical Microbiology, 2015).

As is true for any environmental or opportunistic pathogen the mere finding of an NTM does not establish disease. For NTM, pathogenicity is largely a species-specific trait (Tortoli, 2014). Many of the environmental contaminants are readily identified by species assignment as unlike pathogens, e.g. *M. gordonae* and most of the rapid growers (Table 1). Guidelines issued by ATS/IDSA have summarized the criteria which establish the clinical significance of an NTM (Griffith et al., 2007). These criteria include: (i) species identified, (ii) positive smear microscopy, (iii) detection in multiple patients' specimens, and (iv) clinical history. Discordant results between culture and PCR results in our study were resolved by evaluation of the patients' clinical history and the availability of additional patients' specimens analyzed (Tables S6, S7 and S8 in Supplemental materials). The results show that diagnosis of NTM by culture would have missed 5 patients and that PCR would have missed 5 patients (Fig. 2), which was shown as a non-significant difference by statistical analysis. These results testify that PCR and culture exhibit comparable sensitivity in detecting clinically relevant NTM disease. The significant workload associated with the interpretation of positive genus PCR results – not the least DNA sequence analysis of the amplicon – is compensated by the faster time-to-result (approximately 2 days including decontamination of the sample) in comparison with culture (1–3 weeks, especially for slow growing NTM) and by the prospects to possibly replace resource-costly cultural detection methods in diagnostic mycobacteriology by molecular screening.

In the third part of this study we evaluated the performance of the AID TB resistance line probe assay (LPA) to rapidly identify drug susceptible MTB in clinical samples that were MTB PCR positive. Our results show an excellent performance of the AID TB resistance line probe assay for smear positive samples (respiratory and non-respiratory specimens, Table 2, Table 3). Inherently, the line probe assay as a multiplex PCR is less sensitive than the COBAS™ TaqMan™ MTB PCR. Consequently, of the 438 specimens positive in the MTB PCR assay, only 311 (71.0%) gave an interpretable LPA result. Increasing the number of MTB PCR positive samples subjected to LPA analysis increases the likelihood of an interpretable result. This is possible as the specificity of the LPA is exceptionally high (> 99%). The results of molecular DST of clinical specimens and phenotypic DST on isolates recovered by culture showed an excellent concordance of 98.5%. Overall the data indicate that treatment of drug-susceptible TB can be initiated based on genotypic resistance results.

We observed a number of minor discrepancies when comparing molecular with phenotypic DST (Table 4; Table S10 in Supplemental materials). 2 isolates showed an isolated low-level INH resistance and no mutation in *inhA* or *katG* – neither by LPA nor by gene sequencing. One isolate had a wild-type *rpoB* LPA result but was resistant to rifampicin at 1.0 mg/l, the corresponding deletion of 9 bps in *rpoB* was not detected by the LPA. Given the current limitations of molecular DST and the in part controversial discussions on genotype – phenotype relationships, resistant LPA results should be confirmed phenotypically to identify possible discrepancies, e.g. some mutations are missed in MGIT critical concentration testing, nonsynonymous mutation in LPA targets may result in a false resistance result (Van Deun et al., 2009, Plinke et al., 2011, Sirgel et al., 2012a, Sirgel et al., 2012b, Sirgel et al., 2013, Böttger, 2011, Kaswa et al., 2014). We deliberately decided to use screening for mutations in *inhA*, *katG*, and *rpoB* by LPA as a surrogate marker of any type of drug resistance. Any isolate with a mutation found in *inhA*, *katG*, or *rpoB* is categorized as drug resistant and is suspect of MDR/XDR until ruled out. Based on this interpretation of LPA results, LPA readily separated drug-susceptible wild-type MTB from drug-resistant MTB isolates.

In principle, the screening for any type of drug resistance by searching for mutations in *inhA*, *katG*, and *rpoB* would allow to limit phenotypic DST to isolates with a mutation identified in *inhA*, *katG*, and *rpoB*. Limiting phenotypic DST to isolates with a mutation identified in *inhA*, *katG*, or *rpoB* would miss PZA and EMB monoresistance and INH and RIF resistance mutations not covered by LPA. Available evidence suggests that monoresistance to PZA, EMB, or INH has little, if any, effect on treatment outcome (Cattamanchi et al., 2009, Singla et al., 2002, Yee et al., 2012, Tan et al., 2016). In contrast, resistance to RIF is a major reason for treatment failure and poor clinical outcome (Mitchison and Nunn, 1986, Winston and Mitruka, 2012). We retrospectively analyzed all *M. tuberculosis* isolates from 2007 to 2015 (n = 2616) which are kept in a curated strain collection at the Institute of Medical Microbiology, University of Zurich. For all of these isolates the results of first-line agent critical concentration testing are available as are the results of quantitative drug susceptibility testing for isolates tested resistant at the critical concentration (Springer et al., 2009, Cambau et al., 2015). In addition, all isolates tested as resistant to isoniazid, rifampicin, ethambutol, or pyrazinamide were subjected to LPA. During this period, LPA would have missed 2 cases of isolated PZA resistance, 1 case of isolated EMB resistance, and 7/76 cases of isolated INH resistance (50/76 isolates were high-level isoniazid resistant with MICs > 1.0 mg/l, 26/76 isolates were low-level isoniazid resistant with MICs < 1.0 mg/l; the 7/76 missed cases of isoniazid resistance were all low-level resistant with no mutation detected by LPA in *inhA* or *katG*). None of the 12 cases of isolated RIF resistance and none of the MDR/XDR cases (n = 84) would have been missed.

**Fig. 3:**
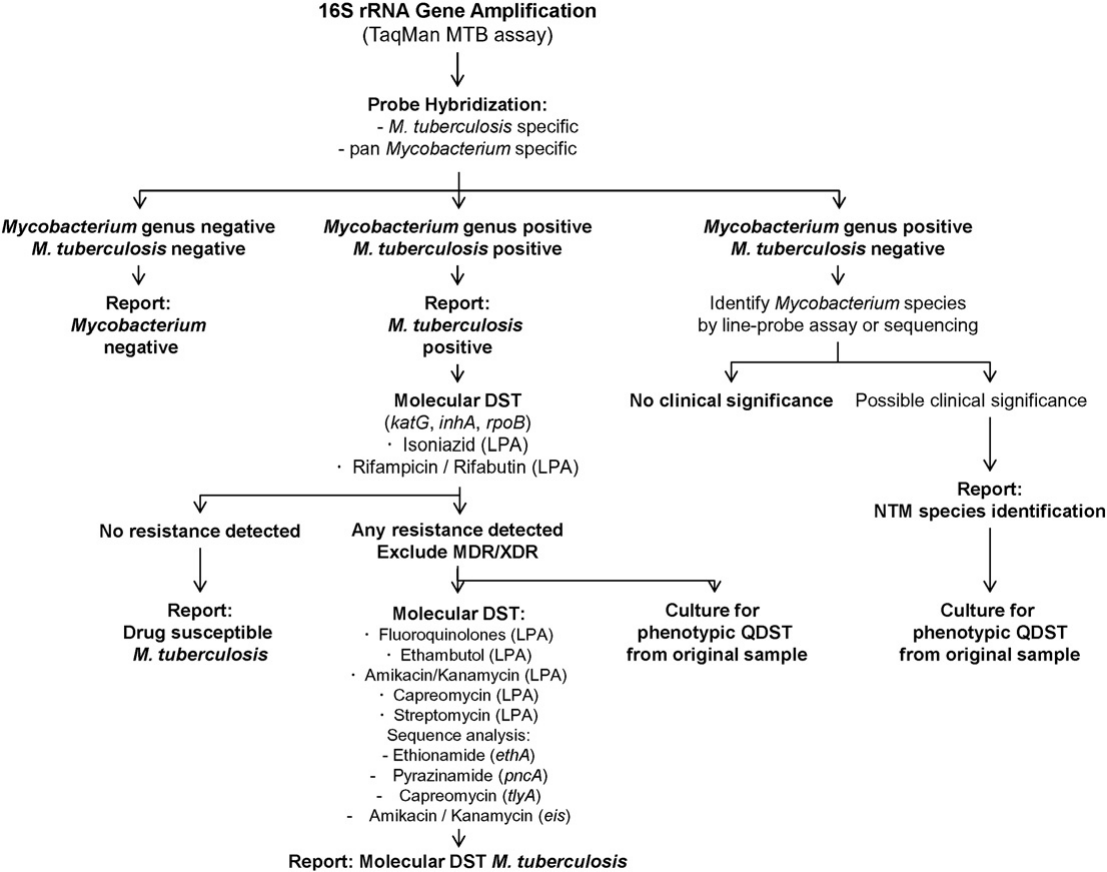
Algorithm for high-throughput molecular diagnostic mycobacteriology.

In summary, by consequently analyzing all clinical samples submitted to our diagnostic mycobacteriology laboratory over a three year period this study demonstrates that molecular diagnostics may largely replace culture for screening of mycobacterial infections and for rapid recognition of drug-susceptible *M. tuberculosis* disease. Based on the results of this study we propose an algorithm to implement high-throughput diagnostic molecular screenings in countries with a low prevalence of *M. tuberculosis* infections (Fig. 3). Samples submitted to the clinical laboratory for the detection of mycobacterial infections, including MTB and NTM, are screened on the basis of 16S rRNA gene amplification. Differentiation between MTB and NTM is made by using two probes, one MTB specific probe and one pan mycobacterial (genus) probe. A positive MTB PCR assay is subsequently complemented by an LPA to detect mutations associated with resistance against first-line drugs (*katG*, *inhA*, *rpoB*). When any mutation is found in *inhA*, *katG* or *rpoB* the specimen is subjected to a full work-up for proper characterization of the isolate's resistance profile, including further LPA, DNA sequencing of target genes, and culture for phenotypic QDST. When identifying a putative novel resistance mutation by DNA sequencing, it is essential to assess the phenotypic QDST results to clarify the role of the mutation – care must be taken not to mix up genetic polymorphisms with resistance mutations (Feuerriegel et al., 2010). A positive NTM screening assay as per the genus probe is followed by sequencing of the PCR amplicon (this study), or alternatively an NTM LPA, to separate clinically relevant NTM from environmental contaminations and NTM with no clinical relevance or closely related genera such as *Corynebacterium*.

As per the diagnostic algorithm, culture is a must for 1. any MTB PCR positive specimen with a resistance mutation identified in *inhA*, *katG* or *rpoB* to allow for full phenotypic QDST and additional epidemiological investigations, 2. any MTB patient where no interpretable LPA has been obtained, 3. any specimen with a clinical relevant NTM to allow for phenotypic DST. Besides this, the algorithm is flexible and the requirements for culture can be adapted as per the laboratories' experience and needs, e.g. defined samples (e.g. biopsies, samples from retreatment cases) or samples with known limited sensitivity for molecular-based tests (e.g. CSF, bone marrow) can be subjected to culture. Culture of MTB PCR positive samples with a susceptible LPA result may not be required when a PCR for differentiation within the *M. tuberculosis* complex is implemented – *Mycobacterium bovis* is naturally resistant to pyrazinamide (Manual of Clinical Microbiology, 2015). A limitation of our study is the little numbers of resistant MTB encountered in the 35 months period. In addition, detection of heteroresistance is a challenge for genetics-based tests (Streicher et al., 2012, FolkvardsenFolkvardsen et al., 2013). Compared to MDR TB which is mostly transmitted (> 95%), de-novo acquisition of resistance during treatment is more frequent for second-line drugs (Streicher et al., 2012).

As long as the discrepancies between phenotypic and molecular DST exist (see above), phenotypic QDST is a mainstay in proper characterization of an isolates' drug susceptibility profile. As is, the speed and easiness of LPAs to screen for a molecular indication of any type of resistance combined with the possibility for a preliminary determination of an isolate's resistance profile where required, is hard to match by any other available technique. Clearly, molecular DST would profit from development of assays as specific and versatile as LPA but as sensitive as single target PCRs, eliminating the need to analyse more than one MTB PCR sample for reliable molecular DST results.

## Funding

This work was supported by the University of Zurich, Switzerland, and the Federal Office of Public Health, Switzerland.

## Conflict of Interest Statement

ECB is a consultant of AID Diagnostika GmbH. The other authors have no conflict of interest to disclose.

## Author Contributions

ECB designed the study. VD, GVB, and PMK collected epidemiological data. CR, AV, and RH did the laboratory work and DNA extraction. VD, GVB, CR, PMK, and ECB interpreted the data, prepared the figures, and did the literature search. VD, GVB, and ECB wrote the report. CR, AV, RH, and PMK commented on the drafts of the paper.

## Figures and Tables

**Table 1:** 16S rRNA gene sequencing assignment of non-tuberculous mycobacteria (NTM).

Concordant identifications from NTM PCR positive and NTM culture positive specimens (n = 191)	NTM from PCR positive and culture negative specimens considered clinically not relevant (n = 130)	NTM from PCR positive and culture negative specimens considered pathogenic NTM (n = 139)	NTM from PCR negative and culture positive specimens
*M. abscessus* complex (n = 57)	*M. gordonae* (n = 36)	*M. abscessus* complex (n = 59)	*M. gordonae* (n = 19)
*M. avium* (n = 35)	*M. gilvum* (n = 17)	*M. avium* (n = 26)	*M. avium* (n = 17)
*M. kansasii*/*gastri* (n = 29)	*M. frederiksbergense* (n = 18)	*M. mucogenicum* (n = 9)	*M. kansasii*/*gastri* (n = 11)
*M. intracellulare* (n = 14)	*M. llatzerense* (n = 16)	*M. kansasii*/*gastri* (n = 8)	*M. xenopi* (n = 10)
*M. chimaera* (n = 14)	*M. phlei* (n = 16)	*M. salmoniphilum* (n = 8)	*M. chimaera* (n = 9)
*M. fortuitum* (n = 7)	*M. neoaurum* complex (n = 15)	*M. fortuitum* (n = 7)	*M. abscessus* complex (n = 7)
*M. xenopi* (n = 12)	*M. vaccae* (n = 4)	*M. xenopi* (n = 4)	*M. fortuitum* (n = 6)
*M. malmoense* (n = 4)	*M. aurum* (n = 2)	*M. malmoense* (n = 2)	*M. interjectum* (n = 4)
*M. gordonae* (n = 4)	*M. chlorophenolicum* (n = 1)	*M. goodii* (n = 2)	*M. senegalense* (n = 3)
*M. marinum*/*ulcerans* (n = 4)	*M. fluoranthenivorans* (n = 1)	*M. chimaera* (n = 1)	*M. conceptionense* (n = 2)
*M. interjectum* (n = 3)	*M. sacrum* (n = 1)	*M. fortuitum* complex (n = 1)	*M. intracellulare* (n = 2)
*M. salmoniphilum* (n = 3)	*M. monacense* (n = 1)	*M. parafortuitum* (n = 1)	*M. marseillense* (n = 2)
*M. vulneris* (n = 3)	*M. murale*/*M. tokaiense* (n = 1)	*M. smegmatis* (n = 1),	*M. celatum* (n = 2)
*M. haemophilum* (n = 1)	*M. rufum* (n = 1)	*M. hassiaticum* (n = 1)	*M. terrae complex* (n = 2)
*M. simiae* (n = 1)		*M. asiaticum* (n = 1)	*M. marinum* (n = 1)
		*M. tusciae* (n = 1)	*M. palustre* (n = 1)
		*M. setense* (n = 1)	*M. porcinum* (n = 1),
		*M. szulgai* (n = 1)	*M. bohemicum* (n = 1)
		Potentially novel slow growing species (n = 5)	*M. colombiense* (n = 1)
			*M. simiae* (n = 1)

**Table 2:** Analysis of COBAS™ TaqMan™ MTB positive specimens (n = 438) by the AID TB resistance line probe assay (LPA) module 1 (isoniazid, rifampicin).

AID test result	Number of specimens								
Respiratory			Non respiratory			Total		
Smear positive	Smear negative	Total	Smear positive	Smear negative	Total	Smear positive	Smear negative	Total
Interpretable	223 (95.3%)	32 (35.6%)	255 (78.7%)	31 (72.1%)	24 (34.3%)	56 (49.1%)	254 (91.7%)	56 (35.0%)	311 (71.0%)
Uninterpretable	11 (4.7%)	58 (64.4%)	69 (21.3%)	12 (27.9%)	46 (65.7%)	58 (50.9%)	23 (8.3%)	104 (65.0%)	127 (29.0%)
Total (100%)	234	90	324	43	70	114	277	160	438

**Table 3:** Analysis of specimens from 253 patients by the AID TB resistance line probe assay module 1 (isoniazid, rifampicin).

AID test result	Number of patients					
Respiratory specimens		Non respiratory specimens		Total	
Total	≥ 3 specimens analyzed	Total	≥ 3 specimens analyzed	Total	≥ 3 specimens analyzed
Interpretable	159 (86.0%)		36 (52.9%)		195 (77.1%)	
Uninterpretable	26 (14.0%)	3/26 (11.5%)	32 (47.1%)	2/26 (7.7%)	58 (22.9%)	5/58 (8.6%)
Total (100%)	185		68		253	

**Table 4:** Comparison of phenotypic (culture) and molecular (AID TB resistance LPA) drug susceptibility test results for isoniazid and rifampicin at patient level (n = 183); at least one MTB isolate for each patient was tested.

		Phenotypic DST (culture)	
resistant	susceptible
Genotypic DST (AID TB resistance LPA)	Rifampicin (RIF; rpoB)		
Resistant	4^a^	–
Susceptible	1^b^	178
		
Isoniazid (IHN; inhA, katG)		
Resistant	9^c^	0
Susceptible	2^d^	172

Cohen's kappa test for agreement: RIF κ = 0.886 and INH κ = 0.894.

a 2/4 isolates showed a RpoB D516V mutation, 1/4 isolates showed a RpoB H526D mutation, and 1/4 isolates showed a RpoB H526L mutation.

b A 9 bps deletion (codon 509–511) was shown in *rpoB*, which is not covered by a probe in the AID TB resistance LPA.

c 1/9 isolates showed a C-15T mutation in the *inhA* promoter and 8/9 showed a KatG S315T mutation; 2 of the 8 KatG S315T mutants showed an additional mutation in *inhA* promoter.

d Isolates showed low level isoniazid resistance (resistant at 0·1 mg/l and susceptible at 1·0 mg/l); mutations in *inhA* or *katG* were not detected by LPA nor by sequencing of *inhA* and *katG.*

## Appendix A Supplementary data

Supplementary material. Image 1
