# Supplementary material for: Diagnostic Molecular Mycobacteriology in Regions With Low Tuberculosis Endemicity: Combining Real-time PCR Assays for Detection of Multiple Mycobacterial Pathogens With Line Probe Assays for Identification of Resistance Mutations
Source: eBioMedicine. 2016 Jun 14;9:228–37. doi: 10.1016/j.ebiom.2016.06.016 (PMC4972562; doi:10.1016/j.ebiom.2016.06.016)
Supplement: Supplementary file 1 — Supplementary material. [file mmc1.docx]

**Supplemental Materials**

Deggim-Messmer V, Bloemberg GV, Ritter C, Voit A, Hömke R, Keller PM, Böttger EC.

**Diagnostic molecular mycobacteriology in regions with low tuberculosis endemicity: combining real-time PCR assays for detection of multiple mycobacterial pathogens with line probe assays for identification of resistance mutations.**

**Table S1.** Sensitivity of the *Mycobacterium* genus probe for the detection of *Mycobacterium tuberculosis* complex (MTBC) in comparison with the MTB probe using the QCMD quality control 2009 for molecular detection of MTB.

| Sample | MTB (chromosomal copies) | Material | **Without** addition of genus probe | |  | **With** addition of genus probe^a^ | |
| --- | --- | --- | --- | --- | --- | --- | --- |
|  |  |  | MTB probe  (CP^b^) | Genus probe  (CP) |  | MTB probe  (CP) | Genus  probe  (CP) |
| 1 | - | PRB^c^ | - | - |  | - | - |
| 2 | 100 | PRB | 37.70 | - |  | 37.47 | 38.78 |
| 3 | 316 | PRB | 35.98 | - |  | 35.56 | 36.53 |
| 4 | 1000 | PRB | 33.98 | - |  | 34.10 | 34.82 |
| 5 | 10000 | PRB | 30.36 | - |  | 30.17 | 31.24 |
|  |  |  |  |  |  |  |  |
| 6 | - | Sputum | - | - |  | - | - |
| 7 | 100 | Sputum | 37.96 | - |  | 37.65 | 39.35 |
| 8 | 316 | Sputum | 36.76 | - |  | 37.01 | 37.92 |
| 9 | 1000 | Sputum | 34.52 | - |  | 34.96 | 35.66 |
| 10 | 10000 | Sputum | 31.75 | - |  | 31.61 | 33.28 |

^a^100 nmol *Mycobacterium* genus probe (in a volume of 1 μl) was added to the COBAS™ TaqMan™ MTB assay with a reaction volume of 50 μl.

^b^CP: crossing point for probe signal.

^c^PRB: protein rich buffer.

Abbreviation: QCMD; Quality Control Molecular Diagnostics (<http://www.qcmd.org/>).

**Table S2.** Detection of non-tuberculous mycobacteria (NTM) using the *Mycobacterium* genus probe integrated into the COBAS™ TaqMan™ MTB assay using chromosomal DNA.

| Culture | Species^a^ | MTB probe  (CP^b^) | Genus probe  (CP) |
| --- | --- | --- | --- |
| 1 | *M. celatum* | - | 38.83 |
| 2 | *M. tusciae* | - | 44.70 |
| 3 | *M. simiae* | - | 43.20 |
| 4 | *M. abscessus* | - | 36.92 |
| 5 | *M. chelonae* | - | 36.73 |
| 6 | *M. fortuitum* | - | 39.11 |
| 7 | *M. triviale* | - | 35.70 |
| 8 | *M. goodii* | - | 34.24 |
| 9 | *M. xenopi* | - | 38.76 |
| 10 | *M. flavescens* | - | 34.98 |

^a^Approximately 1 pg of chromosomal DNA was used.

^b^CP: crossing point for probe signal.

**Table S3.** Detection of non-tuberculous mycobacteria (NTM) by means of the *Mycobacterium* genus probe in clinical specimens culture positive for mycobacteria.

| Specimen number | MTB probe  (CP^a^) | Genus probe (CP^a^) | Sequence identification |
| --- | --- | --- | --- |
| 1 | 33.33 | 32.74 | *M. tuberculosis* complex |
| 2 | 29.67 | 30.63 | *M. tuberculosis* complex |
| 3 | 27.98 | 27.31 | *M. tuberculosis* complex |
| 4 | 36.21 | 37.36 | *M. tuberculosis* complex |
| 5 | - | 25.96 | *M. chelonae / abscessus* complex |
| 6 | - | 30.77 | *M. haemophilum* |
| 7 | - | 30.19 | *M. kansasii* |
| 8 | - | 23.80 | *M. chelonae / abscessus* complex |
| 9 | - | 30.50 | *M. chelonae / abscessus* complex |
| 10 | - | 31.13 | *M. malmoense* |
| 11 | - | 34.99 | *M. malmoense* |
| 12 | - | 31.85 | *M. chelonae / abscessus* complex |
| 13 | - | 29.24 | *M. haemophilum* |
| 14 | - | 26.54 | *M. genavense* |
| 15 | - | 31.91 | *M. kansasii* |
| 16 | - | 28.76 | *M. szulgai* |
|  |  |  |  |
| PC^b^ MTB TaqMan assay | 38.26 | 39.11 |  |

^a^CP: crossing point for probe signal.

^b^PC: positive (internal) control for MTB for the COBAS™ TaqMan™ MTB assay.

**Table S4:** Discrepancy analysis of 25 PCR MTB positive and culture MTB negative clinical specimens. The 25 specimens were isolated from 19 patients having received no TB treatment at time of or shortly before specimen collection (see Figure 1).

| **Patient** | **Specimen** | **MTB PCR** | **MTB culture** | **Smear microscopy** | **Clinical diagnosis** | **Specimens¹ analyzed by PCR** | |  | **Specimens¹ analyzed by culture** | |
| --- | --- | --- | --- | --- | --- | --- | --- | --- | --- | --- |
|  |  |  |  |  |  | **Total number** | **Additional PCR MTB positive specimens** |  | **Total number** | **Culture MTB positive** |
| S4.1 | Br asp | Positive | Negative | Negative | Pulmonary TB | ≥3² | 0 |  | ≥3² | 0 |
| S4.2 | Br asp | Positive | Negative | Negative | Pulmonary TB | ≥3 | 0 |  | ≥3 | 1 |
|  | Sputum | Positive | Negative | Negative |  |  |  |  |  |  |
| S4.3 | Br asp | Positive | Negative | Negative | Pulmonary TB (relapse or reinfection) | ≥3 | 0 |  | ≥3 | 0 |
|  | Lymph node | Positive | Negative | Negative |  |  |  |  |  |  |
| S4.4 | Tissue (lung) | Positive | Negative | Negative | Pulmonary TB | ≥3 | 0 |  | ≥3 | 0 |
| S4.5 | Tissue (lung) | Positive | Negative | Negative | Pulmonary TB | 2 | 0 |  | 2 | 0 |
|  | BAL | Positive | Negative | Negative |  |  |  |  |  |  |
| S4.6 | Lymph node | Positive | Negative | Negative | Pulmonary TB | ≥3 | 2 |  | ≥3 | 2 |
| S4.7 | BAL | Positive | Negative | Positive | Pulmonary TB (relapse or reinfection)) | 1 | 0 |  | ≥3 | 0 |
| S4.8 | Gastric fluid | Positive | Negative | Negative | Pulmonary TB | ≥3 | 0 |  | ≥3 | 1 |
| S4.9 | Lymph node | Positive | Negative | Negative | Lymph node TB | 1 | 0 |  | 1 | 0 |
| S4.10 | Lymph node | Positive | Negative | Negative | Lymph node TB (relapse or reinfection; previous abdominal TB) | ≥3 | 0 |  | ≥3 | 0 |
|  | Lymph node | Positive | Negative | Negative |  |  |  |  |  |  |
|  | Lymph node | Positive | Negative | Positive |  |  |  |  |  |  |
| S4.11 | Lymph node | Positive | Negative | Negative | Lymph node TB (relapse or reinfection; previous pulmonary TB) | 1 | 0 |  | ≥3 | 0 |
| S4.12 | Lymph node | Positive | Negative | Negative | Lymph node TB (relapse or reinfection) | 2 | 0 |  | 2 | 0 |
| S4.13 | Lymph node | Positive | Negative | Negative | Lymph node TB | 1 | 0 |  | 1 | 0 |
| S4.14 | Lymph node | Positive | Negative | Positive | Lymph node TB | 1 | 0 |  | 1 | 0 |
| S4.15 | Lymph node | Positive | Negative | Positive | Lymph node TB | 2 | 0 |  | 2 | 0 |
|  | Lymph node | Positive | Negative | Negative |  |  |  |  |  |  |
| S4.16 | Ear swab | Positive | Negative | Negative | Middle ear TB | ≥3 | 2 |  | ≥3 | ≥3 |
| S4.17 | Sputum | Positive | Negative | Positive | Miliary TB | ≥3 | 2 |  | ≥3 | ≥3 |
| S4.18 | Bone aspirate | Positive | Negative | Positive | TB osteomyelitis | 1 | 0 |  | 1 | 0 |
| S4.19 | Urine | Positive | Negative | Negative | BCG instillation (bladder) | 1 | 0 |  | 1 | 0 |

¹Samples: (i) for pulmonary TB proper specimens considered are: sputum, BAL, brocheal aspirate, gastric fluid in children, pleural fluid, and thoracic lymph node; (ii) for abdominal TB proper specimens considered are: ascitic fluid, tissue peritoneum, tissue omentum, and abdominal lymph node;(iii) for miliary TB all specimens are considered.

²Number of samples analyzed.**Table S5.** Discrepancy analysis of 79 culture MTB positive and PCR MTB negative clinical specimens isolated. The 79 specimens were isolated from 64 patients (see Figure 1).

| **Patient** | **Specimen** | **MTB PCR** | **MTB culture** | **Smear microscopy** | **Clinical diagnosis** |  | **Specimens analyzed by culture** | |  | **Specimens¹ analyzed by PCR** | |
| --- | --- | --- | --- | --- | --- | --- | --- | --- | --- | --- | --- |
|  |  |  |  |  |  |  | **Total number** | **Additional culture MTB positive** |  | **Total number** | **PCR MTB positive** |
| S5.1 | Sputum | Negative | Positive | Negative | Pulmonary TB |  | ≥3 | ≥3 |  | ≥3² | ≥3 |
|  | Sputum | Negative | Positive | Negative |  |  |  |  |  |  |  |
| S5.2 | Br asp | Negative | Positive | Negative | Pulmonary TB |  | ≥3 | 2 |  | 2 | 1 |
| S5.3 | Sputum | Negative | Positive | Negative | Pulmonary TB |  | ≥3 | 1 |  | ≥3 | 1 |
| S5.4 | Br asp | Negative | Positive | Negative | Pulmonary TB |  | 2 | 0 |  | 2 | 0 |
| S5.5 | Br asp | Negative | Positive | Negative | Pulmonary TB |  | 2 | 0 |  | 2 | 0 |
| S5.6 | Tissue (bronchus) | Negative | Positive | Negative | Pulmonary TB |  | ≥3 | 1 |  | ≥3 | 1 |
| S5.7 | Sputum | Negative | Positive | Negative | Pulmonary TB |  | ≥3 | ≥3 |  | 2 | 1 |
| S5.8 | Sputum | Negative | Positive | Negative | Pulmonary TB |  | ≥3 | ≥3 |  | ≥3 | 1 |
|  | Sputum | Negative | Positive | Negative |  |  |  |  |  |  |  |
| S5.9 | Sputum | Negative | Positive | Negative | Pulmonary TB |  | ≥3 | 0 |  | ≥3 | 0 |
|  | BAL | Negative | Positive | Negative |  |  |  |  |  |  |  |
| S5.10 | Br asp | Negative | Positive | Negative | Pulmonary TB |  | ≥3 | 1 |  | ≥3 | 1 |
| S5.11 | Sputum | Negative | Positive | Negative | Pulmonary TB |  | ≥3 | 0 |  | ≥3 | 0 |
| S5.12 | Sputum | Negative | Positive | Negative | Pulmonary TB |  | ≥3 | 0 |  | ≥3 | 2 |
| S5.13 | Sputum | Negative | Positive | Positive | Pulmonary TB |  | ≥3 | 2 |  | ≥3 | 2 |
| S5.14 | Br asp | Negative | Positive | Negative | Pulmonary TB |  | ≥3 | 2 |  | ≥3 | 2 |
| S5.15 | Br asp | Negative | Positive | Negative | Pulmonary TB |  | 2 | 1 |  | 2 | 1 |
| S5.16 | Br asp | Negative | Positive | Negative | Pulmonary TB and Skin TB |  | 2 | 1 |  | 2 | 1 |
| S5.17 | Sputum | Negative | Positive | Negative | Pulmonary TB |  | ≥3 | 0 |  | ≥3 | 0 |
| S5.18 | Br asp | Negative | Positive | Negative | Pulmonary TB |  | 2 | 0 |  | 2 | 0 |
| S5.19 | Br asp | Negative | Positive | Negative | Pulmonary TB |  | 2 | 0 |  | 2 | 0 |
| S5.20 | Sputum | Negative | Positive | Negative | Pulmonary TB |  | ≥3 | 0 |  | ≥3 | 0 |
| S5.21 | Sputum | Negative | Positive | Negative | Pulmonary TB |  | ≥3 | 1 |  | ≥3 | 1 |
| S5.22 | Sputum | Negative | Positive | Negative | Pulmonary TB |  | ≥3 | 0 |  | ≥3 | 1 |
|  | Sputum | Negative | Positive | Negative |  |  |  |  |  |  |  |
| S5.23 | Sputum | Negative | Positive | Negative | Pulmonary TB |  | ≥3 | 2 |  | ≥3 | 1 |
| S5.24 | Br asp | Negative | Positive | Negative | Pulmonary TB |  | ≥3 | 1 |  | 2 | 0 |
| S5.25 | Lymph node | Negative | Positive | Negative | Pulmonary TB |  | ≥3 | ≥3 |  | 2 | 1 |
| S5.26 | Gastric fluid | Negative | Positive | Negative | Pulmonary TB |  | ≥3 | 0 |  | ≥3 | 1 |
| S5.27 | Lymph node | Negative | Positive | Negative | Lymph node TB |  | ≥3 | 1 |  | 2 | 0 |
| S5.28 | Lymph node | Negative | Positive | Negative | Lymph node TB |  | 1 | 0 |  | 1 | 0 |
| S5.29 | Lymph node | Negative | Positive | Negative | Lymph node TB |  | 1 | 0 |  | 1 | 0 |
| S5.30 | Lymph node | Negative | Positive | Negative | Lymph node TB |  | 1 | 0 |  | 1 | 0 |
| S5.31 | Lymph node | Negative | Positive | Negative | Lymph node TB |  | 1 | 0 |  | 1 | 0 |
| S5.32 | Lymph node | Negative | Positive | Negative | Lymph node TB |  | 2 | 1 |  | 2 | 1 |
| S5.33 | Lymph node | Negative | Positive | Negative | Lymph node TB |  | 1 | 0 |  | 1 | 0 |
| S5.34 | Lymph node | Negative | Positive | Negative | Lymph node TB |  | 1 | 0 |  | 1 | 0 |
| S5.35 | Lymph node | Negative | Positive | Negative | Lymph node TB |  | 1 | 0 |  | 1 | 0 |
| S5.36 | Lymph node | Negative | Positive | Negative | Lymph node TB |  | 1 | 0 |  | 1 | 0 |
| S5.37 | Lymph node | Negative | Positive | Negative | Lymph node TB |  | 1 | 0 |  | 1 | 0 |
| S5.38 | Lymph node | Negative | Positive | Negative | Lymph node TB |  | 1 | 0 |  | 1 | 0 |
| S5.39 | Lymph node | Negative | Positive | Negative | Lymph node TB |  | 1 | 0 |  | 1 | 0 |
| S5.40 | Lymph node | Negative | Positive | Negative | Lymph node TB |  | 2 | 1 |  | 1 | 0 |
| S5.41 | Pleural fluid | Negative | Positive | Negative | Pleural TB |  | 2 | 0 |  | 2 | 0 |
|  | Pleural fluid | Negative | Positive | Negative |  |  |  |  |  |  |  |
| S5.42 | Pleural tissue | Negative | Positive | Negative | Pleural TB |  | 2 | 0 |  | 2 | 0 |
| S5.43 | Pleural fluid | Negative | Positive | Negative | Pleural TB |  | 3 | 2 |  | 1 | 0 |
| S5.44 | Pleural fluid | Negative | Positive | Negative | Pleural TB |  | 2 | 1 |  | 2 | 1 |
| S5.45 | Pleural fluid | Negative | Positive | Negative | Pleural TB |  | 2 | 0 |  | 1 | 0 |
| S5.46 | Pleural fluid | Negative | Positive | Negative | Pleural TB |  | 2 | 0 |  | 2 | 0 |
| S5.47 | Pericardial tissue | Negative | Positive | Negative | TB pericarditis |  | 1 | 0 |  | 1 | 0 |
| S5.48 | Br asp | Negative | Positive | Negative | TB Meningitis (miliary TB) |  | ≥3 | 1 |  | ≥3 | 2 |
|  | Br asp | Negative | Positive | Negative |  |  |  |  |  |  |  |
| S5.49 | Ascitic fluid | Negative | Positive | Negative | Miliary TB |  | ≥3 | 2 |  | ≥3 | 1 |
| S5.50 | Sputum | Negative | Positive | Negative | Miliary TB |  | 2 | 1 |  | 2 | 1 |
|  | Tissue liver | Negative | Positive | Negative |  |  |  |  |  |  |  |
| S5.51 | Ascitic fluid | Negative | Positive | Negative | Miliary TB |  | ≥3 | 2 |  | ≥3 | 1 |
|  | Tissue | Negative | Positive | Negative |  |  |  |  |  |  |  |
|  | Sputum | Negative | Positive | Negative |  |  |  |  |  |  |  |
|  | Sputum | Negative | Positive | Negative |  |  |  |  |  |  |  |
| S5.52 | Sputum | Negative | Positive | Negative | Miliary TB |  | ≥3 | ≥3 |  | ≥3 | 3 |
| S5.53 | Tissue omentum | Negative | Positive | Negative | Abdominal TB |  | ≥3 | 0 |  | 1 | 0 |
| S5.54 | Aspirate abdominal | Negative | Positive | Negative | Abdomial TB |  | ≥3 | 1 |  | ≥3 | 1 |
|  | Aspirate abdominal | Negative | Positive | Negative |  |  |  |  |  |  |  |
| S5.55 | Tissue omentum | Negative | Positive | Negative | Abdominal TB |  | 2 | 1 |  | 2 | 1 |
| S5.56 | Ascitic fluid | Negative | Positive | Negative | Abdominal TB |  | ≥3 | 1 |  | ≥3 | 1 |
| S5.57 | Ascitic fluid | Negative | Positive | Negative | Abdominal TB |  | 2 | 1 |  | 2 | 0 |
| S5.58 | Tissue peritoneum | Negative | Positive | Negative | Abdominal TB |  | ≥3 | 2 |  | ≥3 | 2 |
|  | Tissue peritoneum | Negative | Positive | Negative |  |  |  |  |  |  |  |
|  | Tissue peritoneum | Negative | Positive | Negative |  |  |  |  |  |  |  |
|  | Tissue peritoneum | Negative | Positive | Negative |  |  |  |  |  |  |  |
| S5.59 | Biospy bone | Negative | Positive | Negative | TB osteomyelitis |  | 1 | 0 |  | 1 | 0 |
| S5.60 | Tissue joint capsule | Negative | Positive | Negative | TB osteomyelitis |  | ≥3 | 2 |  | 2 | 0 |
|  | Pericardial tissue | Negative | Positive | Negative |  |  |  |  |  |  |  |
| S5.61 | Bone biopsy | Negative | Positive | Negative | TB spondylodiscitis |  | ≥3 | 3 |  | ≥3 | 3 |
| S5.62 | Sputum | Negative | Positive | Negative | TB osteomyelitis |  | ≥3 | ≥3 |  | ≥3 | 1 |
| S5.63 | Tissue ear | Negative | Positive | Negative | Ear TB |  | ≥3 | 2 |  | ≥3 | 3 |
| S5.64 | Superficial wound | Negative | Positive | Negative | Salivary gland TB |  | ≥3 | 0 |  | 2 | 0 |

¹samples: (i) for pulmonary TB proper specimens considered are: sputum, BAL, bronchial aspirate, gastric fluid in children, pleural fluid, and thoracic lymph node; (ii) for abdominal TB proper specimens considered are: ascitic fluid, tissue peritoneum, tissue omentum, and abdominal lymph node;(iii) for miliary TB all specimens are considered.

**Table S6.** Analysis of 11 PCR NTM positive specimens with possible clinical relevance for which sequence analysis of the PCR product resulted in assignment of a NTM that was different from the NTM recovered by culture. These 11 samples were collected from 8 patients (see Figure 2).

| **Patient** | **Specimen** | **PCR** |  | **Culture** |  | **Smear microscopy** | **Clinical diagnosis** | **Samples analyzed by PCR** | |  | **Samples analyzed by culture** | |
| --- | --- | --- | --- | --- | --- | --- | --- | --- | --- | --- | --- | --- |
|  |  |  |  |  |  |  |  | **Total Number** | **Additional identification of corresponding NTM by PCR** |  | **Total Number** | **Additional identification of corresponding NTM by culture** |
| S6.1 | Sputum | *M. abscessus* complex | **✓** | *M. avium* | **✓** | Positive | Cystic fibrosis | 1 | 0 |  | ≥3 | 2 |
| S6.2 | Sputum | *M. abscessus* complex | **✓** | *M. kansasii* | **✓** | Negative | Bronchiectasis | ≥3 | 2 |  | ≥3 | 0 |
| S6.3 | Bone marrow | *M. genavense* | **✓** | *M. avium* | **✓** | ND | Disseminated  *M. avium* and  *M. genavense* infection | 1 | 0 |  | ≥3 | 0 |
| S6.4 | Sputum | *M. llatzerense* | **-** | *M. avium* | **✓** | Negative | Disseminated *M. avium* infection (HIV positive) | 2 | 0 |  | ≥3 | 2 |
| S6.5 | Sputum | *M. genavense* | **✓** | *M. avium* | **✓** | Positive | Disseminated *M. avium* and *M. genavense* infection (HIV positive) | ≥3 | 0 |  | ≥3 | 0 |
|  | Sputum | *M. genavense* | **✓** | *M. avium* | **✓** | Negative |  |  |  |  |  |  |
|  | Sputum | *M. genavense* | **✓** | *M. avium* | **✓** | Positive |  |  |  |  |  |  |
|  |  |  |  |  |  |  |  |  |  |  |  |  |
| S6.6 | Sputum | *M. avium* | *(***✓)** | *M. avium* and  *M. kansasii* | *(***✓)** | Positive | unclear | 2 | 0 |  | ≥3 | 0 |
|  | Sputum | *M. avium* | *(***✓)** | *M. avium* and  *M. abscessus* complex | *(***✓)** | Positive |  |  |  |  |  |  |
| S6.7 | Sputum | *M. salmoniphilum* | **-** | *M. chimaera* | **✓** | Negative | Pulmonary infiltrate | ≥3 | 0 |  | ≥3 | 0 |
| S6.8 | Sputum | *M. malmoense* | **✓** | *M. gordonae* | **-** | Negative | COPD | ≥3 | 0 |  | ≥3 | 1 |

^1^clinical relevance: **✓** yes; **-** no; *(***✓**) most likely clinical relevant (no clinical data available)

**Table S7.** Discrepancy analysis of 48 culture negative specimens for which a positive COBAS™ TaqMan™ *Mycobacterium* genus PCR with sequence identification of a possibly clinically relevant NTM was obtained (see Figure 2). The specimens were obtained from 21 patients.

| **Patient** | **Specimen** | **Genus PCR** | **16S gene sequence result** | **Culture** | **Smear microscopy** | **Disease related to mycobacteria** | **Samples analyzed by PCR** | |  | **Samples analyzed by culture** | |
| --- | --- | --- | --- | --- | --- | --- | --- | --- | --- | --- | --- |
|  |  |  |  |  |  |  | **Total Number** | **Additional PCR positive  (identical NTM)** |  | **Total Number** | **Culture positive (identical NTM)** |
| S7.1 | Br asp | Positive | *M. kansasii/*  *gastri* | Negative | Positive | Pulmonary cavern | 2 | 1 |  | ≥3 | ≥3 |
| S7.2 | Sputum | Positive | *M. kansasii*  */gastri* | Negative | Negative | Bronchiectasis | ≥3 | 0 |  | ≥3 | 1 |
| S7.3 | Sputum | Positive | *M. abscessus* complex | Negative | Negative | Cystic fibrosis | ≥3 | 1 |  | ≥3 | 1 |
|  | Sputum | Positive | *M. abscessus* complex | Negative | Negative |  |  |  |  |  |  |
|  | Sputum | Positive | *M. abscessus* complex | Negative | Negative |  |  |  |  |  |  |
| S7.4 | Sputum | Positive | *M. abscessus* complex | Negative | Negative | Cystic fibrosis | ≥3 | 1 |  | ≥3 | ≥3 |
| S7.5 | Sputum | Positive | *M. xenopi* | Negative | Negative | Pulmonary infiltrate | ≥3 | 2 |  | ≥3 | ≥3 |
| S7.6 | Sputum | Positive | *M. kansasii/*  *gastri* | Negative | Positive | Pulmonary cavern | 2 | 1 |  | ≥3 | ≥3 |
| S7.7 | Br asp | Positive | *M. chimaera* | Negative | Negative | Disseminated NTM infection | ≥3 | 2 |  | ≥3 | ≥3 |
| S7.8 | Lymph node | Positive | *M. abscessus* complex | Negative | Negative | Cystic fibrosis | ≥3 | ≥3 |  | ≥3 | ≥3 |
|  | Seroma puncture | Positive | *M. abscessus* complex | Negative | Negative |  |  |  |  |  |  |
|  | Deep wound | Positive | *M. abscessus* complex | Negative | Negative |  |  |  |  |  |  |
|  | Deep wound | Positive | *M. abscessus* complex | Negative | Negative |  |  |  |  |  |  |
|  | Superficial wound | Positive | *M. abscessus* complex | Negative | Negative |  |  |  |  |  |  |
|  | Pus | Positive | *M. abscessus* complex | Negative | Negative |  |  |  |  |  |  |
|  | Deep wound | Positive | *M. abscessus* complex | Negative | Positive |  |  |  |  |  |  |
|  | Tissue | Positive | *M. abscessus* complex | Negative | Negative |  |  |  |  |  |  |
| S7.9 | Sputum | Positive | *M. fortuitum* | Negative | Negative | Relapsing lung infection | ≥3 | 1 |  | ≥3 | ≥3 |
| S7.10 | Pleural fluid | Positive | *M. abscessus* complex | Negative | Negative | Cystic fibrosis | ≥3 | ≥3 |  | ≥3 | ≥3 |
|  | Fluid (drain?) | Positive | *M. abscessus* complex | Negative | Negative |  |  |  |  |  |  |
| S7.11 | Sputum | Positive | *M. abscessus* complex | Negative | Negative | Bronchiectasis | ≥3 | 1 |  | ≥3 | ≥3 |
| S7.12 | Sputum | Positive | *M. abscessus* complex | Negative | Negative | Bronchiectasis | ≥3 | 0 |  | ≥3 | 1 |
| S7.13 | Sputum | Positive | *M. abscessus* complex | Negative | Negative | Cystic fibrosis | ≥3 | 0 |  | ≥3 | 0 |
|  | Sputum | Positive | *M. abscessus* complex | Negative | Negative |  |  |  |  |  |  |
| S7.14 | Sputum | Positive | *M. abscessus* complex | Negative | Negative | Cystic fibrosis | ≥3 | ≥3 |  | ≥3 | ≥3 |
|  | Sputum | Positive | *M. abscessus* complex | Negative | Negative |  |  |  |  |  |  |
| S7.15 | Sputum | Positive | *M. abscessus* complex | Negative | Negative | Cystic fibrosis | ≥3 | ≥3 |  | ≥3 | ≥3 |
|  |  |  |  |  |  |  |  |  |  |  |  |
|  |  |  |  |  |  |  |  |  |  |  |  |
| S7.16 | Sputum | Positive | *M. abscessus* complex | Negative | Negative | Bronchiectasis | ≥3 | 0 |  | ≥3 | 0 |
|  | Sputum | Positive | *M. abscessus* complex | Negative | Negative |  |  |  |  |  |  |
|  | Sputum | Positive | *M. abscessus* complex | Negative | Negative |  |  |  |  |  |  |
|  | Sputum | Positive | *M. abscessus* complex | Negative | Negative |  |  |  |  |  |  |
|  | Sputum | Positive | *M. abscessus* complex | Negative | Negative |  |  |  |  |  |  |
|  | Sputum | Positive | *M. abscessus* complex | Negative | Negative |  |  |  |  |  |  |
|  | Sputum | Positive | *M. abscessus* complex | Negative | Positive |  |  |  |  |  |  |
|  | Sputum | Positive | *M. abscessus* complex | Negative | Negative |  |  |  |  |  |  |
|  | Sputum | Positive | *M. abscessus* complex | Negative | Negative |  |  |  |  |  |  |
|  | Sputum | Positive | *M. abscessus* complex | Negative | Negative |  |  |  |  |  |  |
|  | Sputum | Positive | *M. abscessus* complex | Negative | Negative |  |  |  |  |  |  |
|  | Sputum | Positive | *M. abscessus* complex | Negative | Negative |  |  |  |  |  |  |
|  | Sputum | Positive | *M. abscessus* complex | Negative | Negative |  |  |  |  |  |  |
| S7.17 | Sputum | Positive | *M. abscessus* complex | Negative | Positive | Bronchiectasis | ≥3 | 0 |  | ≥3 | 1 |
|  | Sputum | Positive | *M. abscessus* complex | Negative | Negative |  |  |  |  |  |  |
| S7.18 | Sputum | Positive | *M. malmoense* | Negative | Negative | COPD | ≥3 | 1 |  | ≥3 | 1 |
|  | Sputum | Positive | *M. malmoense* | Negative | Negative |  |  |  |  |  |  |
| S7.19 | Sputum | Positive | *M. abscessus* complex | Negative | Negative | Cystic fibrosis | ≥3 | 0 |  | ≥3 | 0 |
|  | Sputum | Positive | *M. abscessus* complex | Negative | Negative |  |  |  |  |  |  |
| S7.20 | Sputum | Positive | *M. abscessus* complex | Negative | Negative | Cystic fibrosis | 1 | 0 |  | 2 | 0 |
| S7.21 | Sputum | Positive | M. avium | Negative | Negative | Cystic fibrosis | ≥3 | 0 |  | ≥3 | 0 |

**Table S8.** Discrepancy analysis of 29 PCR NTM negative specimens for which a possibly clinically relevant NTM was recovered by culture (see Figure 2). The specimens corresponded to 18 patients.

| **Patient** | **Specimen** | **Genus PCR** | **Culture** | **Smear micros-copy** | **Disease related to mycobacteria** | **Samples analyzed by culture** | |  | **Samples analyzed by PCR** | |
| --- | --- | --- | --- | --- | --- | --- | --- | --- | --- | --- |
|  |  |  |  |  |  | **Total Number** | **Additional positive (identical NTM)** |  | **Total Number** | **PCR positive (identical NTM)** |
| S8.1 | BAL | Negative | *M. xenopi* | Positive | Pulmonary infiltrate | ≥3 | 2 |  | ≥3² | 2 |
| S8.2 | Br asp | Negative | *M. kansasii / gastri* | Negative | Bronchiectasis | ≥3 | 0 |  | ≥3 | 1 |
| S8.3 | Lymph node | Negative | *M. avium* | Negative | Pulmonary infiltrate | ≥3 | ≥3 |  | ≥3 | ≥3 |
|  | BAL | Negative | *M. avium* | Negative |  |  |  |  |  |  |
| S8.4 | Sputum | Negative | *M. abscessus* complex | Negative | Cystic fibrosis | ≥3 | 2 |  | ≥3 | 2 |
|  | Sputum | Negative | *M. abscessus* complex | Negative |  |  |  |  |  |  |
|  | Sputum | Negative | *M. abscessus* complex | Negative |  |  |  |  |  |  |
| S8.5 | Sputum | Negative | *M. xenopi* | Negative | Pulmonary infiltrate | ≥3 | 2 |  | ≥3 | 3 |
|  |  |  |  |  |  |  |  |  |  |  |
| S8.6 | Sputum | Negative | *M. chimaera* | Negative | Disseminated infection | ≥3 | ≥3 |  | ≥3 | 2 |
|  | Urine | Negative | *M. chimaera* | Negative |  |  |  |  |  |  |
| S8.7 | Sputum | Negative | *M. avium*  *M. interjectum* | Negative | COPD | ≥3 | 0 |  | ≥3 | 0 |
|  | Sputum | Negative | *M. avium*  *M. interjectum* | Negative |  |  |  |  |  |  |
|  | Sputum | Negative | *M. avium* | Negative |  |  |  |  |  |  |
|  | Sputum | Negative | *M. interjectum* | Negative |  |  |  |  |  |  |
| S8.8 | Sputum | Positive (MTB^+^) | *M. tuberculosis*  *M. fortuitum* | Positive | Mixed pulmonary TB / *M. fortuitum* infection | ≥3 | ≥3 |  | 2 | 1 (genus^+^, MTB^+^) |
| S8.9 | Sputum | Negative | *M. fortuitum* | Negative | Lung infection | ≥3 | 1 |  | ≥3 | 2 |
|  | Sputum | Negative | *M. fortuitum* | Negative |  |  |  |  |  |  |
|  |  |  |  |  |  |  |  |  |  |  |
| S8.10 | BAL | Negative | *M. avium* | Negative | Disseminated infection | ≥3 | ≥3 |  | ≥3 | 0 |
|  | Br asp | Negative | *M. avium* | Negative |  |  |  |  |  |  |
| S8.11 | Sputum | Negative | *M. fortuitum* | Negative | COPD | ≥3 | 1 |  | ≥3 | 1 |
| S8.12 | BAL | Negative | *M. kansasii / gastri* | Negative | COPD | 2 | 1 |  | 2 | 1 |
| S8.13 | Sputum | Negative | *M. abscessus* complex | Negative | Bronchiectasis | ≥3 | 2 |  | ≥3 | 2 |
| S8.14 | Bronchial aspiration | Negative | *M. intracellulare* | Negative | Bronchiectasis | ≥3 | ≥3 |  | ≥3 | 2 |
| S8.15 | Biopsy skin | Negative | *M. marinum* | Negative | Swimming pool granuloma | 1 | 0 |  | 1 | 0 |
| S8.16 | Sputum | Negative | *M. abscessus* complex | Negative | Cystic fibrosis | ≥3 | ≥3 |  | ≥3 | ≥3 |
|  | Sputum | Negative | *M. abscessus* complex | Negative |  |  |  |  |  |  |
|  | Sputum | Negative | *M. abscessus* complex | Negative |  |  |  |  |  |  |
| S8.17 | Sputum | Negative | *M. avium* | Negative | Pulmonary cavern | ≥3 | 1 |  | ≥3 | 1 |
| S8.18 | Biopsy knee | Negative | *M. conceptionense* | Negative | Abscess | 2 | 0 |  | 1 | 0 |

**Table S9.** Discrepancy analysis of 3 discrepant results comparing the AID TB resistance line probe assay module 1 and conventional phenotypic DST

|  | **AID LPA result** | | | | | | |  | **Phenotypic DST result** | | |  |
| --- | --- | --- | --- | --- | --- | --- | --- | --- | --- | --- | --- | --- |
|  | **specimen** | | |  | **culture** | | |  | **culture** | | |  |
| **Specimen number** | ***inhA***^a^ | ***katG***^b^ | ***rpoB***^c^ |  | ***inhA*** | ***katG*** | ***rpoB*** |  | **INH**  **0.1 mg/L** | **INH**  **1.0 mg/L** | **RIF**  **1.0 mg/L** | **Further genetic analysis** |
| 2011176091 | WT | WT | WT |  | WT | WT | WT |  | R | S | S | *inhA* gene and promoter sequencing: WT |
| 2012179742 | WT | WT | WT |  | WT | WT | WT |  | R | S | S | *inhA* gene and promoter sequencing: WT |
| 2012176662 | WT | MUT | WT |  | WT | MUT | WT |  | R | R | R | *rpoB* 81 bp gene sequencing: 9 bps deletion (codon 509-511) |

Abbreviations: DST: drug susceptibility testing; LPA: line probe assay; INH: isoniazid; RIF: rifampicin; WT: wild-type; MUT: mutation; S: susceptible; R: resistant; ND: not determined; bp: base pair.

^a^Probes on the LPA: INH wild-type (*inhA* -16, -15, -8) and INH mut. (*inhA* -16G, -15T, -8A, -8C)

^b^Probes on the LPA: INH wild-type (KatG 315) and INH mut. (KatG S315T)

^c^Probes on the LPA: RIF wild-type (RpoB 513-516), RIF mut. (RpoB D516V, D516Y), RIF wild-type (RpoB 522-526), RIF mut. (RpoB H526Y. H526D, H526R), RIF wild-type (RpoB 529-533), RIF mut. (RpoB S531L, S531W)

**Table S10.** Quantitative phenotypic drug susceptibility testing^a^ results and resistance-associated mutations identified by line-probe assays and sequencing of amplified gene fragments for all isolates with mutations in *katG*, *inhA* and/or *rpoB.*

| Isolate number | | | 10186016 | 10177162 | 11186003 | 11186006/  11186014 | 11179812 | 12186103/  12176662 | 12186112 | 12179950 | 12180743 |
| --- | --- | --- | --- | --- | --- | --- | --- | --- | --- | --- | --- |
| Specimen |  |  | BAL | Sputum | Tissue | Sputum | Lymph node | Sputum | Tracheal bronchial aspirate | Aspirate | Lymph node |
| **INH (mg/L)** | **0.1** | | R | R | R | R | R | R | R | R | R |
|  | 1 | | R | R | R | R | R | R | R | R | R |
|  | 3 | | R | R | R | R | S | R | S | R | R |
|  | 10 | | S | S | R | R | S | S | S | S | S |
| *inhA* promoter |  | | WT | WT | mut | mut | C-15T | WT | WT | WT | WT |
| *katG* |  | | S315T | S315T | S315T | S315T | WT | S315T | S315T | S315T | S315T |
| **ETH (mg/L)** | **5** | | S | S | R | R | R | R | R | S | S |
|  | 10 | | S | S | R | R | R | S | S | S | S |
|  | 25 | | S | S | R | R | R | S | S | S | S |
| *ethA* |  | | WT | nd | WT | WT | WT | bp 28ins: frameshift | W167S, S266R | nd | nd |
| **RIF (mg/L)** | **1** | | R | S | R | R | S | R | R | S | S |
|  | 4 | | R | S | R | S | S | S | R | S | S |
|  | 20 | | R | S | R | S | S | S | S | S | S |
| **RBT (mg/L)** | 0.1 | | R | S | R | S | S | S | R | S | S |
|  | **0.4** | | R | S | S | S | S | S | S | S | S |
|  | 2 | | R | S | S | S | S | S | S | S | S |
| *rpoB* |  | | H526D | WT | D516V | H526L | WT | 9 bp deletion; codon 509-511 | D516V | WT | WT |
| **EMB (mg/L)** | **5** | | R | S | S | R | S | S | S | S | S |
|  | 12.5 | | S | S | S | S | S | S | S | S | S |
|  | 50 | | S | S | S | S | S | S | S | S | S |
| *embB* |  | | M306I | WT | WT | M306V | WT | WT | M306V | WT | WT |

| Isolate number | | | 10186016 | 10177162 | 11186003 | 11186006/  11186014 | 11179812 | 12186103/  12176662 | 12186112 | 12179950 | 12180743 |
| --- | --- | --- | --- | --- | --- | --- | --- | --- | --- | --- | --- |
| Specimen |  |  | BAL | Sputum | Tissue | Sputum | Lymph node | Sputum | Tracheal bronchial aspirate | Aspirate | Lymph node |
| **PZA (mg/L)** | **100** | | S | S | S | S | S | S | R | S | S |
| Pyrazinamidase |  | | positive | nd | positive | positive | nd | positive | negative | nd | nd |
| *pncA* |  | | nd | nd | WT | WT | nd | WT | insertion  (IS6110 element  at bp 226) | nd | nd |
| **STR (mg/L)** | **1** | | R | nd | R | R | R | R | R | nd | nd |
|  | 4 | | S | nd | R | R | R | S | R | nd | nd |
|  | 20 | | S | nd | R | R | R | S | S | nd | nd |
| *rspL* |  | | WT | WT | WT | WT | K43R | WT | K88R | WT | WT |
| *rrs* (SM mutations) |  | | WT | WT | A523C^b^ | A523C^b^ | WT | WT | WT | WT | G524C^b^ |
| **AMK (mg/L)** | **1** | | S | nd | S | R | nd | S | S | S | nd |
|  | 4 | | S | nd | S | S | nd | S | S | S | nd |
|  | 20 | | S | nd | S | S | nd | S | S | S | nd |
| **CAP (mg/L)** | **2.5** | | S | nd | S | R | nd | S | S | nd | nd |
|  | 5 | | S | nd | S | R | nd | S | S | nd | nd |
|  | 25 | | S | nd | S | S | nd | S | S | nd | nd |
| *rrs(16S rRNA)* |  | | WT | WT | WT | WT | WT | WT | WT | WT | WT |
| *tlyA* |  | | WT | nd | nd | del bp 102-121 | nd | nd | nd | nd | nd |
| **MOX (mg/L)** | **0.25** | | S | nd | S | S | nd | S | R | S | S |
|  | 0.5 | | S | nd | S | S | nd | S | S | S | S |
|  | 2.5 | | S | nd | S | S | nd | S | S | S | S |
|  | 7.5 | | S | nd | S | S | nd | S | S | S | S |
| *gyrA* |  | | WT | WT | WT | WT | WT | WT | WT | WT | WT |

^a^Quantitative drug susceptibility testing was done using the BACTEC MGIT 960 / TB eXiST system (Becton-Dickinson Inc., East Rutherford, NJ, USA).

Critical concentrations of first- and second-line antituberculosis drugs in the MGIT 960 system are highlighted in bold underlined. TB: tuberculosis. Nd: not determined.
Line probe assay results from clinical specimens are given. LPA results were complemented by nucleic acid sequence analysis.

^b^Numbering of rRNA position according to *E. coli* (for the corresponding *M. tuberculosis* position substract 10).

**Drug abbreviations:** INH, isoniazid; RIF, rifampicin; RBT, rifabutin; EMB, ethambutol; PZA, pyrazinamide; STR, streptomycin; AMK, amikacin; CAP, capreomycin; MOX, moxifloxacin.
